# What is the Prevalence of Low Health Literacy in European Union Member States? A Systematic Review and Meta-analysis

**DOI:** 10.1007/s11606-020-06407-8

**Published:** 2021-01-05

**Authors:** V. Baccolini, A. Rosso, C. Di Paolo, C. Isonne, C. Salerno, G. Migliara, G. P. Prencipe, A. Massimi, C. Marzuillo, C. De Vito, P. Villari, F. Romano

**Affiliations:** 1 Department of Public Health and Infectious Diseases, Sapienza University of Rome, Rome, Italy; 2 Harvard TH Chan School of Public Health, Boston, MA USA; 3 Local Health Unit Roma 2, Rome, Italy

**Keywords:** health literacy, prevalence, systematic review, meta-analysis, Europe

## Abstract

**Background:**

Many studies have shown that low health literacy (HL) is associated with several adverse outcomes. In this study, we systematically reviewed the prevalence of low HL in Europe.

**Methods:**

PubMed, Embase, and Scopus were searched. Cross-sectional studies conducted in the European Union (EU), published from 2000, investigating the prevalence of low HL in adults using a reliable tool, were included. Quality was assessed with the Newcastle-Ottawa Scale. Inverse-variance random effects methods were used to produce pooled prevalence estimates. A meta-regression analysis was performed to assess the association between low HL and the characteristics of the studies.

**Results:**

The pooled prevalence of low HL ranged from of 27% (95% CI: 18–38%) to 48% (95% CI: 41–55%), depending on the literacy assessment method applied. Southern, Western, and Eastern EU countries had lower HL compared to northern Europe (*β*: 0.87, 95% CI: 0.40–1.35; *β*: 0.59, 95% CI: 0.25–0.93; and *β*: 0.72, 95% CI: 0.06–1.37, respectively). The assessment method significantly influenced the pooled estimate: compared to word recognition items, using self-reported comprehensions items (*β*: 0.61, 95% CI: 0.15–1.08), reading or numeracy comprehensions items (*β*: 0.77, 95% CI: 0.24–1.31), or a mixed method (*β*: 0.66, 95% CI: 0.01–1.33) found higher rates of low HL. Refugees had the lowest HL (*β*: 1.59, 95% CI: 0.26–2.92). Finally, lower quality studies reported higher rates of low HL (*β*: 0.56, 95% CI: 0.06–1.07).

**Discussion:**

We found that low HL is a public health challenge throughout Europe, where one in every three to almost one in every two Europeans may not be able to understand essential health-related material. Additional research is needed to investigate the underlying causes and to develop remedies.

**PROSPERO Registration:**

CRD42019133377

**Supplementary Information:**

The online version contains supplementary material available at 10.1007/s11606-020-06407-8.

## INTRODUCTION

There is a growing interest among public health professionals and policy makers in health literacy (HL), which can be broadly defined as “[people’s ability] to make judgements and take decisions in everyday life concerning healthcare, disease prevention and health promotion to maintain or improve their quality of life”^1^. Low literacy is a worldwide phenomenon^2^: limited or non-adequate HL is associated with increased hospitalization^3,4^, higher rates of medication non-adherence^3,5^, lower uptake of preventive interventions^3^, poorer overall health status and increased mortality in the elderly^5^, as well as an increase in healthcare costs^6^. Furthermore, low literacy follows a social gradient and reinforces existing inequalities^7^.

Given its health effects, several European Union (EU) initiatives address HL: in 2007, HL was identified as a policy priority in the European Commission’s health strategy “Together for Health 2007-2013”^8^; in 2012, improving HL was included among the priorities of the Health 2020 strategy of the World Health Organization (WHO) Regional Office for Europe^9^; and in 2014, the first comparative survey on population literacy across eight EU countries was conducted^10^. At the international level, the WHO included HL as one of the key health promotion pillars needed for a successful 2030 Agenda for Sustainable Development ^11^.

The number of studies on HL has escalated in recent years^12^; these studies have shown that most patient education material, including explanations of health services and their benefits, are often incomprehensible to a significant proportion of people^13^. However, small sample sizes, narrowly defined patient populations and heterogeneity in outcomes or study designs have limited the generalizability of the results^14^, limiting its usefulness for policymaking^15^. Within this context, we conducted a systematic review and meta-analysis of cross-sectional studies to quantify the prevalence of low HL in adult people living in EU countries, to provide a quantitative synthesis and estimation of its magnitude at national and European level, and to improve the understanding of the underlying predictive factors.

## METHODS

This study was performed according to the *Cochrane Handbook for Systematic Reviews* and the Preferred Reporting Items for Systematic Reviews and Meta-Analyses (PRISMA) statement^16,17^. The review protocol was registered at PROSPERO (CRD42019133377).

### Search Strategy, Study Selection, and Inclusion Criteria

Three reviewers searched the bibliographic databases PubMed, Embase, and Scopus using the following string: (“health literacy”[Title/Abstract]) AND (((evaluat*[Title/Abstract]) OR measure*[Title/Abstract]) OR assess*[Title/Abstract]). The string was adapted to fit the search criteria of each database (Supplementary Table 1). No reference librarian was involved. The worldwide discussion on a comprehensive HL definition started at the beginning of the twenty-first century^18^; therefore, all articles published between 1 January 2000 and 23 June 2019 were retrieved, without restrictions of language or paper type. The search was supplemented by scanning the reference lists of the relevant articles.

Duplicate articles were removed, and the title and abstract of all retrieved records were screened. Studies that did not meet the inclusion criteria were excluded. Full texts of potentially relevant articles were examined by three researchers and reasons for exclusion were recorded. As suggested by Jackson et al.^19^, we used Google Translate to assess for inclusion of non-English and non-Italian-language articles.

We included any article with the following characteristics: (i) cross-sectional design; (ii) conducted in one or more European Union countries (EU-28); (iii) quantified the prevalence of low HL using a valid and reliable tool; (iv) included people aged 18 years or over.

We excluded articles that investigated only specific HL (e.g., oral HL) that assessed only specific HL domains without providing a general measurement or that did not report the prevalence of low HL in its target population(s).

### Data Collection and Quality Assessment

For each record, three reviewers independently extracted the following information: first author, year of publication, tool used to quantify the prevalence of people with low HL, assessment method, number of items of the tool, proportion of people with low HL, sample size, country, EU geographic area, target population, mean or median age of the sample. Supplementary Table 2 illustrates the characteristics of the HL instruments that were used to assess HL and the cut-off scores that were considered to identify people with low HL. The assessment method was classified according to the structure of the tool in four different categories: using word recognition items, using reading or numeracy comprehension items, using self-reported comprehension items, or using a mixed method (i.e., combination of self-reported and reading or numeracy comprehension items). Countries were grouped as North, East, West, and South Europe, according to the United Nations classification^20^. The target population was classified as general population (i.e., without specific characteristics reported), oncology patients, chronic disease patients, or refugees.

Three independent authors performed the quality assessment of the articles included in the systematic review using the Newcastle-Ottawa Scale for evaluating cross-sectional/survey studies^21^. Articles were considered of high quality when the total score was ≥ 7, fair quality if the score was ≥ 5 and < 7, and poor quality if the score was lower than 5^22^.

### Statistical Analysis

Since most articles provided two or more prevalence estimates (e.g., in different populations, in different age groups, in different countries, using different tools), we considered each estimate to be a different estimate. They will hereafter be collectively referred to as “studies.”

As in a few reports the same target population was investigated using more than one HL tool, separate meta-analyses were conducted. Specifically, we performed an inverse-variance weighted meta-analysis using a logit transformation of the proportions for each HL assessment method. We pooled estimates using a random effects approach^23^ and the restricted maximum likelihood method^24^. The *I*^2^ metric was used to test for heterogeneity^25^.

A random effects meta-regression analysis using logit-transformed prevalence was performed to explore the association between study characteristics and pooled low HL prevalence^25,26^. The robust variance estimation was used to take into account the correlation between studies^27^. We ran univariate and multivariable analyses including the covariates that could influence the prevalence estimate based on literature review. The final model consisted of the following variables: geographical area, study quality, assessment method, target population, and mean/median of the sample. The category with the highest number of studies was used as reference for geographic region, target population, and study quality; for the assessment method, we used the category yielding the lowest illiteracy pooled estimate; for the age groups, we followed the natural gradient, using the youngest as reference. For the breakdown of the age categories, we used the cut-off values reported in most studies. All analyses were performed using STATA (StataCorp), version 16.0.

## RESULTS

### Study Selection

**Figure 1:**
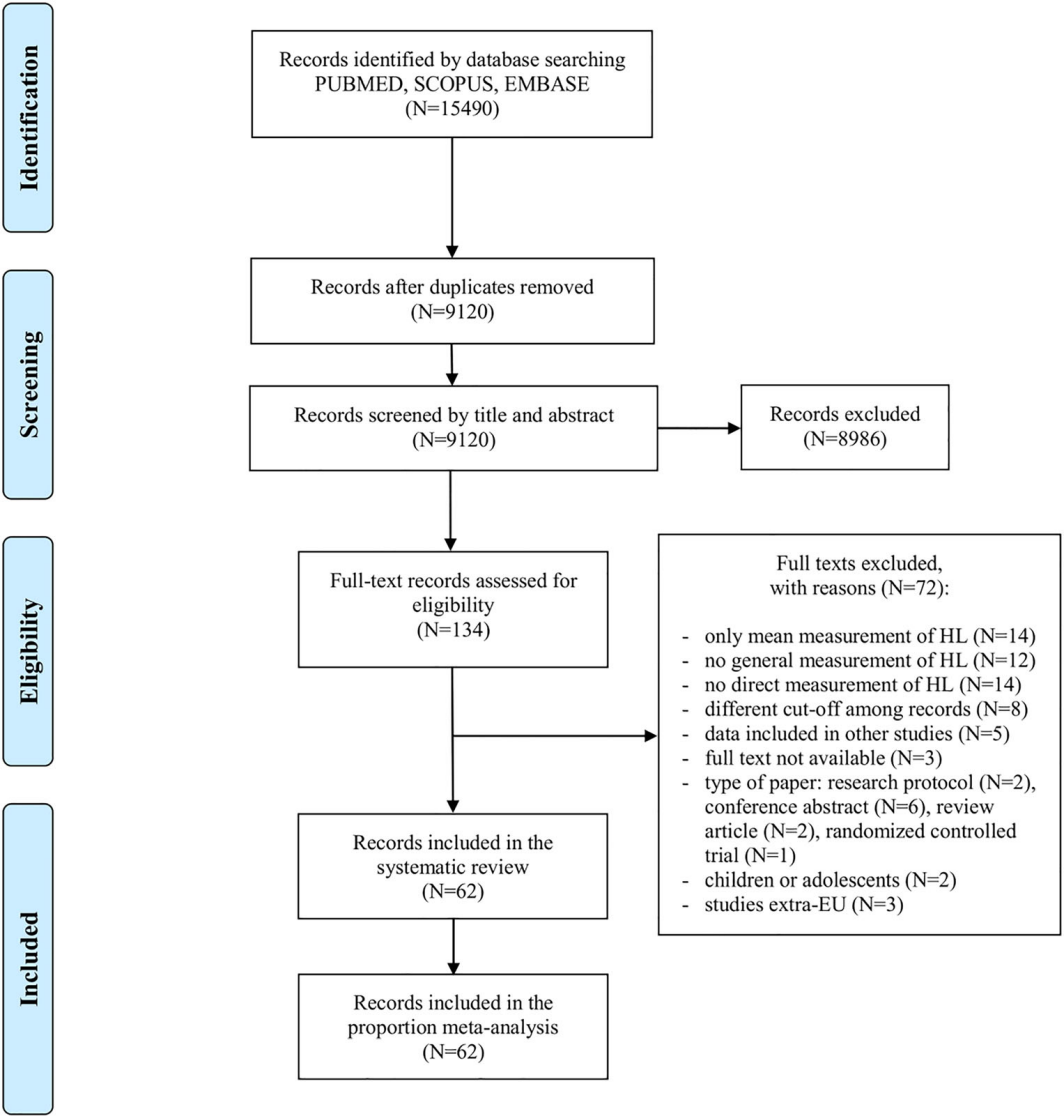
PRISMA flow diagram of the review process. EU, European Union.

After removal of duplicates, 9120 records resulted from the systematic search (Fig. 1). Screening by title and abstract yielded 134 articles that were then assessed for eligibility. A total of 62 articles were ultimately included in this systematic review, which provided the data of 101 studies (Supplementary Table 3). Since in two reports^28,29^ the same target population was investigated by different tools but applying the same HL assessment method, only the prevalence estimate coming from the most frequently used tool was included in the meta-analysis, for a total of 99 studies that were pooled.

### Characteristics of the Studies Included in the Proportion Meta-analysis

A similar number of studies was available from countries of the North^10,30–53^, South^10,54–71^, and West EU region^10,28,72–85^ (Table 1). Conversely, only four studies were conducted in the East^10,86,87^, and two studies referred to refugees from non-EU countries^29,88^. A consistent heterogeneity was observed among the tools used to assess HL: the most frequently used tools were the Newest Vital Sign, applied in 21 studies^42,43,45,47,52,55–58,63,67,73,77,79,82,84^, followed by the European Health Literacy Survey Questionnaire with 16 items (*n* = 15)^26,27,33,34,38,50,60,71,75,78,83,86^ and the Rapid Estimate of Adult Literacy in Medicine (*n* = 12)^30,31,40–42,47–49,73,76^. The European Health Literacy Survey Questionnaire with 47 items^38,43,55,62,63,78,84,87^ and its longer version with 86 items^10,58^ were used eleven and nine times each, respectively, followed by the Single Item Literacy Screener (*n* = 8)^44,61,65,66,69,82^ and the Medical Term Recognition Test (*n* = 7)^56,64,65^ (Table 1). The other tools were used in a limited number of studies, from one to three each (Table 1). Most studies investigated HL using self-reported comprehension items^26,27,30,33,34,36,38,44,48–50,54,55,58,60,61,67,68,70–72,74–76,78,81,83,85,86^ (*n* = 38); 29 studies used reading or numeracy comprehension items^35,37,39–43,45–47,51,52,55–58,63,67,73,77,79,80,82,84^; 23 studies used word recognition items^28–32,58,59,62,65,66,79,82^; and nine studies used a mixed method^10,58^. Sixty-six studies quantified low HL in the general population^10,26,29,31–34,36,39,40,43–45,47,48,50–65,70,71,73,75,76,78,80,83,84^. Patients with chronic diseases were investigated in 25 studies^28,35,37,38,41,42,46,49,66,68,69,71,74,77,79,82,85^, whereas a smaller number of studies (*n* = 6) looked at oncology patients^47,61,75,80^. Only two studies^29,88^ investigated low HL in refugees. Among the 79 studies reporting it, the age of the sample varied, from 45 years or less in 17 studies^30,43,49,56,57,59,64,69,78^; between 46 and 64 years in 47 studies^26,29,30,34,36–38,40,42–45,49–51,54–57,59,63,67,70,71,73,75,79–82,84,85^; and over 64 years in 15 studies^31,33,35,39,41,46,62,66,68–70,72,74,77,78^. Lastly, the vast majority of studies (*n* = 81) were rated as high quality^10,27,29–35,38–40,42,43,45–52,55–58,62–81,83–86^. Eighteen studies were judged as being of fair or poor quality^26,28,36,37,41,44,53,54,59–61,82^; their main deficits were a lack of justification for the sample size and a lack of comparability between participants and non-participants (data not shown).

**Table 1:** Characteristics of the Studies Included in the Proportion Meta-analysis of Low Health Literacy (HL) in European Union (EU) Member States

	*N* (%)
Geographic EU region	
North Europe	33 (33.3)
South Europe	32 (32.3)
West Europe	28 (28.3)
East Europe	4 (4.1)
Refugees coming from non-EU countries	2 (2.0)
HL tool	
NVS	21 (21.3)
HLS-EU-Q16	15 (15.2)
REALM	12 (12.1)
HLS-EU-Q47	11 (11.1)
HLS-EU-Q86	9 (9.1)
SILS	8 (8.1)
METER	7 (7.1)
SBSQ single item	3 (3.0)
SAHLSA-50	3 (3.0)
TOFHLA	3 (3.0)
IALS tool	2 (2.0)
BSAIT	1 (1.0)
TOFHLA-short	1 (1.0)
SAHLPA-23	1 (1.0)
HALS	1 (1.0)
S-FHL	1 (1.0)
HL assessment method	
Self-reported comprehension items	38 (38.4)
Reading or numeracy comprehension items	29 (29.3)
Word recognition items	23 (23.2)
Mixed	9 (9.1)
Target population	
General population	66 (66.7)
Chronic disease patients	25 (25.3)
Oncology patients	6 (6.0)
Refugees coming from non-EU countries	2 (2.0)
Mean or median age	
≤ 45 years	17 (17.1)
46–64 years	47 (47.5)
≥ 65 years	15 (15.2)
Not reported	20 (20.2)
Study quality	
High quality	81 (81.8)
Poor–fair quality	18 (18.2)

*NVS* Newest Vital Sign, *HLS-EU-Q* European Health Literacy Survey Questionnaire, *REALM* Rapid Estimate of Adult Literacy in Medicine, *SILS* Single Item Literacy Screener, *METER* Medical Term Recognition Test, *SBSQ* Set of Brief Screening Questions, *SAHLSA* Short Assessment of Health Literacy for Spanish Adults, *TOFHLA* Test of Functional Health Literacy in Adults, *IALS* International Adult Literacy Survey, *BSAIT* Basic Skill Assessment Initial Test, *SAHLPA* Short Assessment of Health Literacy in Portuguese Adults, *HALS* Health Activities Literacy Scale, *S-FHL* Scale for Functional Health Literacy

### Proportion Meta-analysis of Low Health Literacy by Country

Overall, the pooled prevalence of low literacy varied, depending on the assessment method used. Among tools with self-reported comprehension items, low HL was present in 42% (95% CI: 36–48%; df = 37, *Q* = 3451.2, *I*^2^ = 99.5%); reading or numeracy comprehension items was 42% (95% CI: 33–53%; df = 28, *Q* = 2471.8, *I*^2^ = 99.4%); word recognition items was 27% (95% CI: 18–38%; df = 22, *Q* = 975.5, *I*^2^ = 98.3%); and mixed methods provided a pooled estimate of 48% (95% CI: 41–55%; df = 8, *Q* = 328.0, *I*^2^ = 97.7%) (Table 2 and Supplementary Figures 1, 2, 3, and 4).

**Table 2:** Pooled Prevalence Estimates (PEs) and Their 95% Confidence Interval (CI) of Low Health Literacy in European Union Member States According to Different Assessment Methods

Overall	Self-reported comprehension items		Reading or numeracy comprehension items		Word recognition items		Mixed method	
	*N*	PE (95% CI)	*N*	PE (95% CI)	*N*	PE (95% CI)	*N*	PE (95% CI)
	38	0.42 (0.36–0.48)	29	0.42 (0.33–0.53)	23	0.27 (0.18–0.38)	9	0.48 (0.41–0.55)
Austria							1	0.56 (0.53–0.59)
Belgium	1	0.41 (0.40–0.42)						
Bulgaria							1	0.62 (0.59–0.65)
Croatia			1	0.58 (0.48–0.67)				
Czech Republic	1	0.44 (0.35–0.53)						
Denmark	2	0.44 (0.32-0.58)	2	0.44 (0.35–0.54)				
Finland	1	0.36 (0.31–0.42)						
France	2	0.51 (0.34–0.67)						
Germany	12	0.44 (0.38–0.51)					1	0.46 (0.43–0.49)
Greece	1	0.54 (0.45–0.63)					1	0.45 (0.42–0.48)
Hungary			1	0.41 (0.35–0.46)				
Ireland	1	0.65 (0.46–0.81)	4	0.41 (0.21–0.65)	5	0.19 (0.17–0.22)	1	0.40 (0.37–0.43)
Italy	3	0.42 (0.33–0.51)	3	0.38 (0.35–0.41)	3	0.72 (0.32–0.93)	1	0.54 (0.51–0.57)
Lithuania	1	0.33 (0.30–0.36)						
Poland							1	0.45 (0.41–0.48)
Portugal	1	0.50 (0.48–0.52)	5	0.29 (0.06–0.73)	6	0.21 (0.08–0.46)		
Spain	3	0.71 (0.47–0.87)	1	0.43 (0.34–0.52)	2	0.33 (0.06–0.80)	1	0.58 (0.55–0.61)
Sweden	1	0.39 (0.36–0.43)	1	0.21 (0.14–0.30)				
The Netherlands	2	0.14 (0.12–0.15)	6	0.68 (0.53–0.79)	3	0.19 (0.16–0.23)	1	0.29 (0.26–0.32)
UK	4	0.16 (0.12–0.20)	5	0.28 (0.17–0.43)	4	0.21 (0.09–0.43)		
Refugees	2	0.65 (0.62–0.69)						

*N* number of studies

There was variation in the number of studies in each country as well as the assessment method used. Austria^10^, Belgium^45^, Bulgaria^10^, Croatia^68^, Czech Republic^87^, Finland^50^, Hungary^86^, Lithuania^43^, and Poland^10^ had data available only from one study each. Low HL was common: Austria, 56% (95% CI: 53–53-59%60%); Belgium, 41% (95% CI: 40–42%); Bulgaria, 62% (95% CI: 59–65%); Croatia, 58% (95% CI: 48–67%); Czech Republic, 44% (95% CI: 35–53%); Finland, 36% (95% CI: 31–42%), Hungary, 41% (95% CI: 35–46%); Lithuania, 33% (95% CI: 30–36%); and Poland, 45% (95% CI: 41–48%). Other EU countries had more than one study, often with different assessment tools. Denmark^32,35,51,52^ low HL was around 44%, in both the assessment methods used (95% CI: 32–58%, df = 1, *Q* = 12.5, *I*^2^ = 92.0%, and 95% CI: 35–54%, df = 1, *Q* = 0.1, *I*^2^ = 0.0%, respectively) (Table 2). France^28,82^ low HL was 51% (95% CI: 34–67%). Germany^10,72,77–79,83,84^ ranged from 44% (95% CI: 38–51%, df = 11, *Q* = 681.4, *I*^2^ = 98.9%) to 46% (95% CI: 43–49%); Greece^10,71^ was 45% (95% CI: 42–48%) to 54% (95% CI: 45–63%). In Ireland^10,30,37,38,41,42,49^, the pooled estimates varied between 19% (95% CI: 17–22%, df = 4, *Q* = 7.6, *I*^2^ = 46.8%), 40% (95% CI: 37–43%), 41% (95% CI: 21–65%; df = 3, *Q* = 194.9, *I*^2^ = 98.4%), and 65% (95% CI: 46–81%). Italy low HL pooled estimates were by self-reported comprehension items^61,65,66^ 42% (95% CI: 33–51%; df = 2, *Q* = 12.4, *I*^2^ = 84.5%); by reading or numeracy comprehension items^61,66,67^, 38% (95% CI: 35–41%; df = 2, *Q* = 1.5, *I*^2^ = 0.0%); by word recognition items^64,65^, 72% (95% CI: 32–93%; df = 2, *Q* = 70.8, *I*^2^ = 97.9%); and by mixed method^58^, 54% (95% CI: 51–57%). As for Portugal^54,55,57,59,70^, the low HL prevalence estimates varied between 21% (95% CI: 8–46%, df = 5, *Q* = 86.1, *I*^2^ = 96.5%), 29% (95% CI: 6–73%, df = 4, *Q* = 139.1, *I*^2^ = 98.2%), and 50% (95% CI: 48–52%). In Spain^10,60,63,69,89^, the prevalence estimates of low HL were, in increasing order, 33% (95% CI: 6–80%, df = 1, *Q* = 55.8, *I*^2^ = 98.2%), 43% (95% CI: 34–52%), 58% (95% CI: 55–61%), and 71% (95% CI: 47–87%). In Sweden^36,53^, low HL ranged from 21% (95% CI: 14–30%) to 39% (95% CI: 36–43%). As for The Netherlands^10,73–76,80,81,85^, the highest pooled prevalence was 68% (95% CI: 53–79%; df = 5, *Q* = 101.0, *I*^2^ = 98.5%), followed by 29% (95% CI: 26–32%), 19% (95% CI: 16–23%; df = 2, *Q* = 2.5, *I*^2^ = 0.0%), and 14% (95% CI: 12–15%; df = 1, *Q* = 0.3, *I*^2^ = 0.0%). The UK^31,33,39,40,44,46–48,90^ had relatively low pooled estimates, varying between 16% (95% CI: 12–20%, df = 3, *Q* = 48.5, *I*^2^ = 92.4%), 21% (95% CI: 9–43%, df = 3, *Q* = 50.8, *I*^2^ = 97.2%), and 28% (95% CI: 17–43%, df = 4, *Q* = 337.2, *I*^2^ = 99.4%). Lastly, the refugees’ population was investigated in two studies^29,88^ with a low HL pooled estimate of 65% (95% CI: 62–69%, df = 1, *Q* = 2.6, *I*^2^ = 60.7%).

### Meta-regression Analysis

Meta-regression analysis found that the geographic region, assessment method, target population, and study quality impacted the results (Table 3). Western, Southern, and Eastern countries had higher rates of low HL compared to Northern EU countries. Lower quality studies found higher rates of low HL. There was no difference in literacy by the population assessed (chronic disease, oncology) or the age of the patient. Refugees had the lowest HL.

**Table 3:** Multivariable Meta-regression Model Predicting the Pooled Estimate of the Prevalence of Low Health Literacy in European Union (EU) Member States

Variables included in the model	Meta-regression coefficient (95% CI)	SE	*P* value
Geographic EU region			
North Europe (*N* = 33)	Ref.		
South Europe (*N* = 32)	0.87 (0.40 to 1.35)	0.23	0.001
West Europe (*N* = 28)	0.59 (0.25 to 0.93)	0.17	0.001
East Europe (*N* = 4)	0.72 (0.06 to 1.37)	0.25	0.038
HL assessment method			
Word recognition items (*N* = 23)	Ref.		
Self-reported comprehension items (*N* = 38)	0.61 (0.15 to 1.08)	0.23	0.011
Reading or numeracy comprehension items (*N* = 29)	0.77 (0.24 to 1.31)	0.26	0.006
Mixed (*N* = 9)	0.66 (0.01 to 1.33)	0.31	0.049
Target population			
General population (*N* = 66)	Ref.		
Oncology patients (N=6)	− 1.22 (− 2.50 to 0.05)	0.46	0.056
Chronic disease patients (N=25)	− 0.08 (− 0.48 to 0.32)	0.20	0.680
Refugees (*N* = 2)	1.59 (0.26 to 2.92)	0.30	0.036
Age category			
≤45 years (*N* = 17)	Ref.		
46–64 years (*N* = 47)	0.01 (− 0.62 to 0.64)	0.30	0.976
≥ 65 years (*N* = 15)	0.64 (− 0.11 to 1.40)	0.36	0.091
Not reported (*N* = 20)	0.16 (− 0.63 to 0.96)	0.37	0.667
Study quality			
High quality (*N* = 81)	Ref.		
Fair–poor quality (*N* = 18)	0.56 (0.06 to 1.07)	0.24	0.031

*CI* confidence interval, *SE* standard error, *HL* health literacy

## DISCUSSION

We found that a third to nearly half of Europeans had low HL. This suggests that a significant percentage of people living in EU may have difficulties in getting access to prevention and healthcare services due to limitations in navigation, comprehension, and decision-making^7^. Although this proportion is slightly lower than that reported by a systematic review of studies on the US population^91^, where nearly one in two had low HL, and is lower than the mean prevalence of 55% reported in Southeast Asian countries^92^, our review confirms that low HL also represents a public health challenge in Europe^12^.

While the prevalence varies considerably by country and the HL assessment method, it seemed to follow a geographic distribution, with the northern countries having lower prevalence than the other EU counterparts. It is possible that the intersection between culture, literacy, and HL may at least partially explain such a difference. Social and cultural context, which includes education, is inextricably linked to how citizens perceive and act on health information^93^. Countries with the lowest prevalence of low HL also have greater years of education^94^ and higher socio-economic status^95^, an important factor in HL^96^. However, specific future research is needed in order to better investigate the causes of such inequality and appropriately assess their impact on HL.

A widely accepted definition of HL is still under discussion^1,97^; accordingly, when the researchers used different assessment methods to explore specific HL skills, the prevalence estimates varied significantly. Notably, apart from Italy, tools with word recognition items tended to provide lower illiteracy prevalence estimates, suggesting that investigating HL as medical vocabulary may underestimate the prevalence. Therefore, although the development and acceptance of a universal measure of HL is challenging, a common definition and a comprehensive instrument for its evaluation would enable a more precise estimation of the magnitude of the problem and a better comparison of evidence^97^.

Older age is reported to be associated with a higher risk of low HL^90,91^. While we found a slightly increasing, although not significant, trend of low HL prevalence across age groups, the combination of incomplete data and heterogeneity of the measures applied may have limited the reliability of this covariate. However, since older age is known to be associated with an increase in health needs and low HL could impair access to healthcare services^14,98,99^, the potential effect of aging on HL should not be overlooked.

Differing cultural and educational backgrounds among patients and providers may result in different attitudes and beliefs, which might influence HL and impair access to healthcare services^88,93,100–102^. It was therefore not surprising that we found the strongest association with low HL in refugees, where the lack of knowledge of the healthcare services of the host country, different cultural conceptions, and the language barrier are probably the main drivers of the HL gap^103^. Since HL is most likely to improve when the messaging and delivery are tailored to the specific needs of individuals and populations^97^, it is imperative that healthcare systems become more culturally and linguistically competent, so that they are able to address the growing diversity among their target populations^13^.

Lastly, the study quality was found to be a significant predictor of the prevalence of low HL. Therefore, as reported by WHO Action Network on Measuring Population and Organizational Health Literacy^15^, more high-quality studies are needed in order to properly understand the extent of the challenge and ensure the generalizability of the results. In particular, our study found that more attention should be paid to how the sample is selected, with regard to the justification of sample size and to demonstrating comparability between responders and non-responders.

To the best of our knowledge, this is the first quantitative synthesis of data on prevalence of low HL in EU countries that enabled a comparison between member States. Nevertheless, it is important to acknowledge the limitations of our study. First, since our objective was to quantify the prevalence of low HL, we included only studies with a cross-sectional design. Second, we excluded articles that used an arbitrary cut-off to identify people with low HL, which provided only a mean measurement of HL, or which analyzed only specific sub-domains. Third, HL tools and target populations were consistently heterogeneous; however, separate analyses and a meta-regression were carried out.

In conclusion, low HL is very common in the EU, where at least one in every three people may not be able to understand essential health-related material. Despite a few variations in the prevalence estimate due to the instrument applied, our results are consistent in showing that low HL represents a public health challenge throughout Europe. Additional efforts to increase the evidence on the underlying causes, to identify areas for intervention, and to implement health practices that effectively address a low level of HL are needed.

## Supplementary Information

ESM 1 (DOCX 1146 kb)
