# Supplementary material for: What is the Prevalence of Low Health Literacy in European Union Member States? A Systematic Review and Meta-analysis
Source: J Gen Intern Med. 2021 Jan 5;36(3):753–61. doi: 10.1007/s11606-020-06407-8 (PMC7947142; doi:10.1007/s11606-020-06407-8)
Supplement: Supplementary file 1 — (DOCX 1146 kb) [file 11606_2020_6407_MOESM1_ESM.docx]

Supplementary Figure 1. Proportion meta-analysis of the prevalence of low health literacy in EU Member States by self-reported comprehension items.


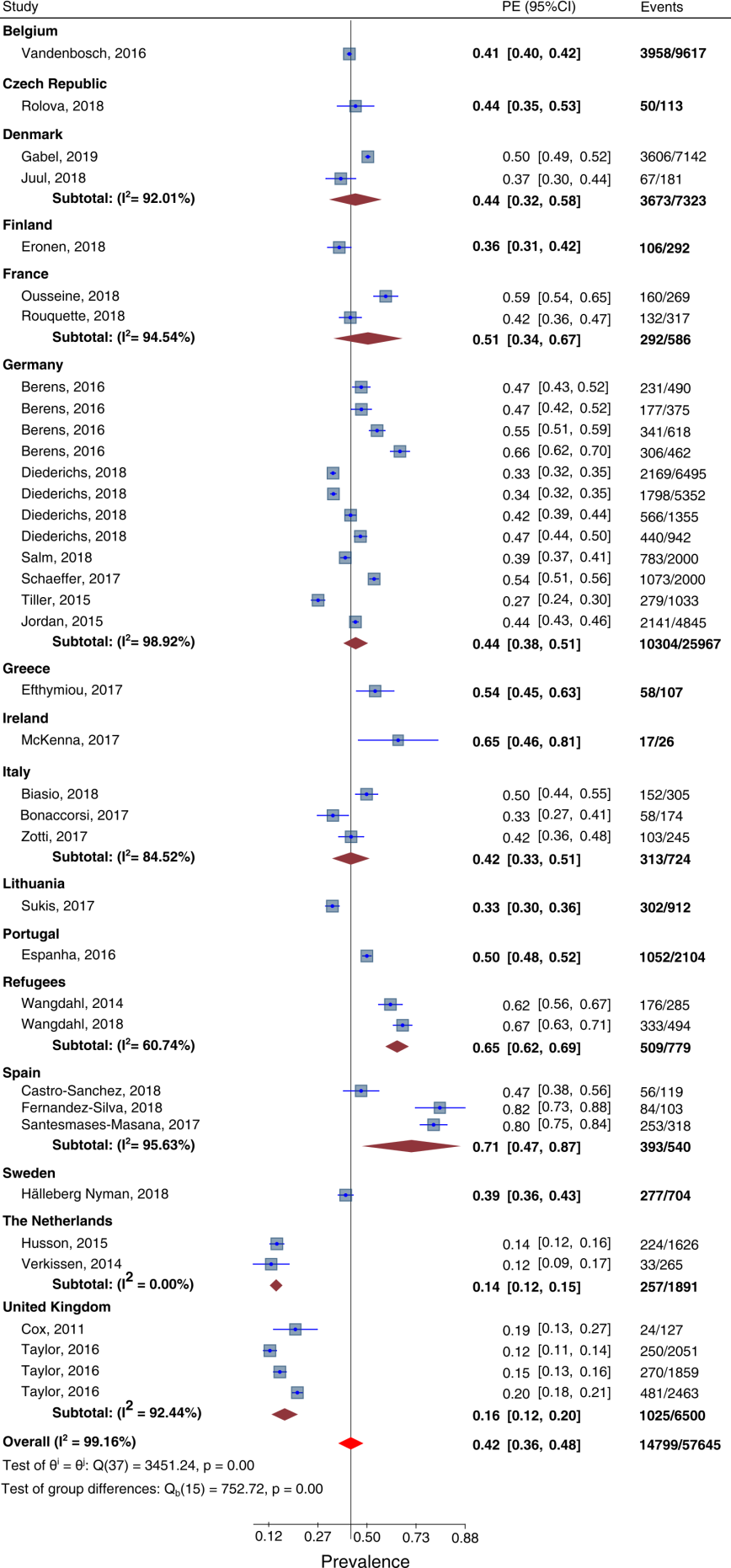


Supplementary Figure 2. Proportion meta-analysis of the prevalence of low health literacy in EU Member States by reading or numeracy comprehension items.


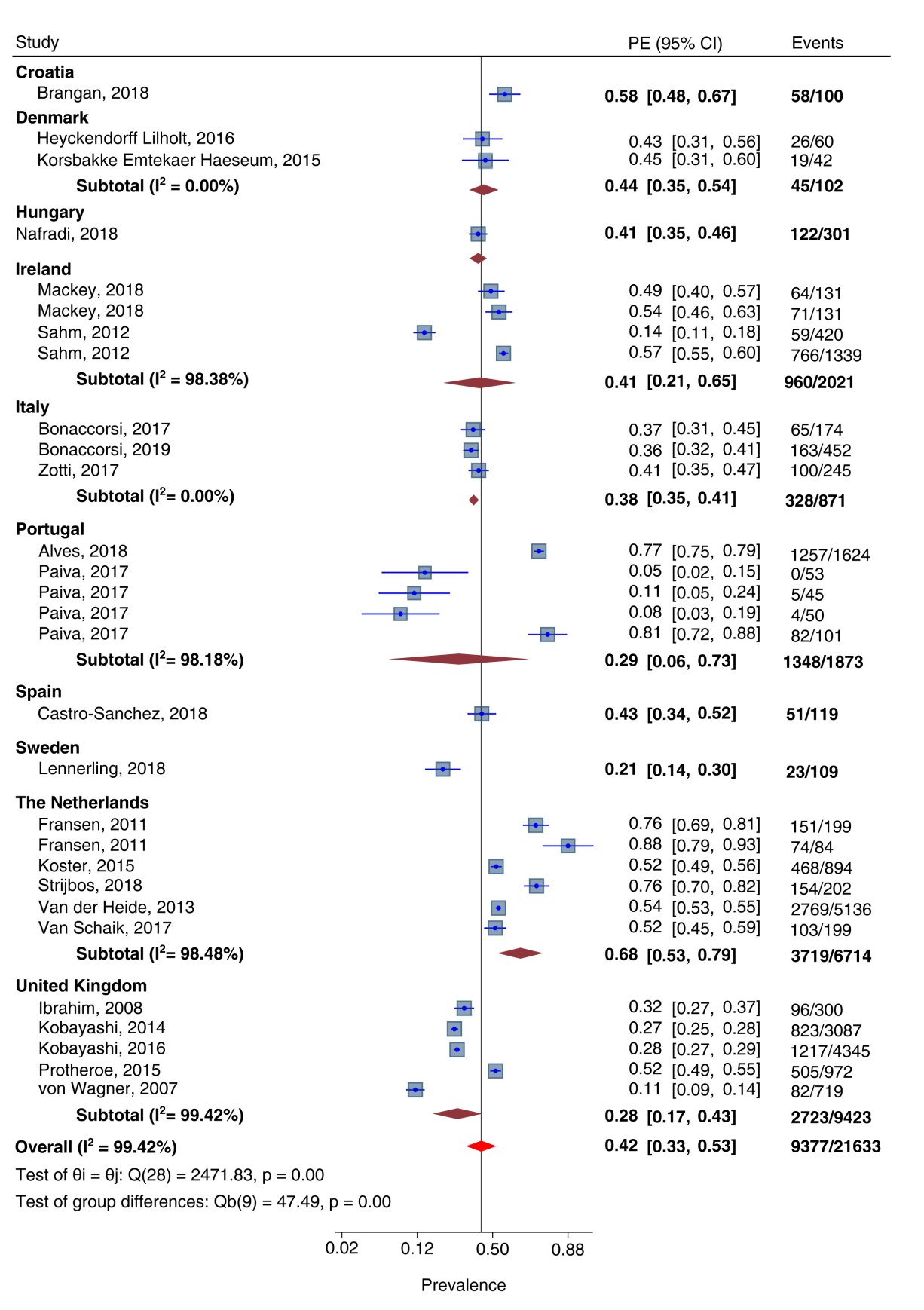


Supplementary Figure 3. Proportion meta-analysis of the prevalence of low health literacy in EU Member States by word recognition items.


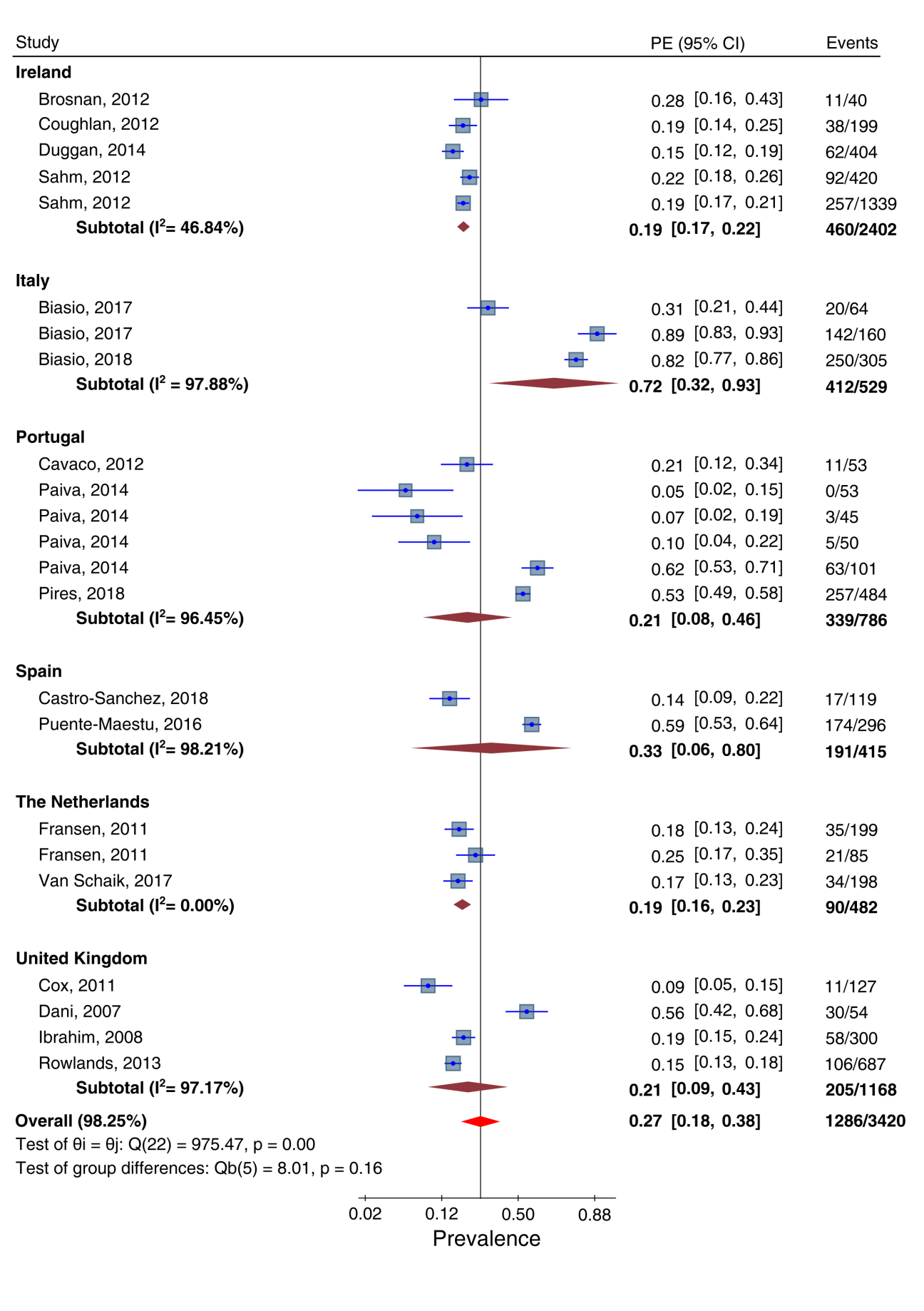


Supplementary Figure 4. Proportion meta-analysis of the prevalence of low health literacy in EU Member States by mixed method.


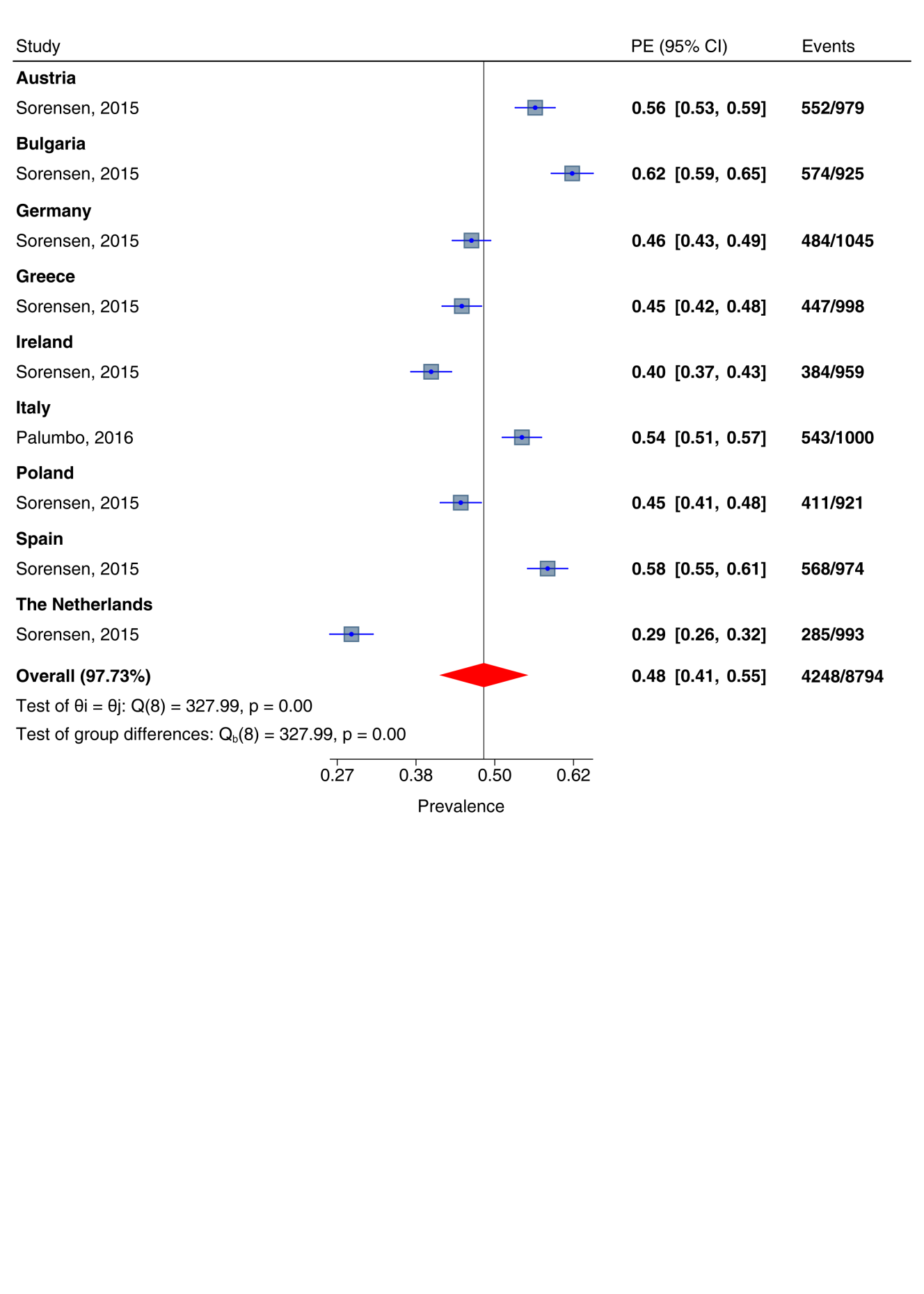


**Appendices**

Supplementary Table 1. Search strategies used in the systematic review.

| (“health literacy”[Title/Abstract]) AND (((evaluat*[Title/Abstract]) OR measure*[Title/Abstract]) OR assess*[Title/Abstract])  Filter: Publication date from 2000/01/01 | PubMed |
| --- | --- |
| health:ab AND literacy:ab AND (evaluat*:ab OR measure*:ab OR assess*:ab) AND [embase]/lim AND [2000-2019]/py | Embase |
| (“health literacy”) AND (((evaluat*) OR measure*) OR assess*) for title/abstract/keywords  Limit: after 2000 | Scopus |

Supplementary Table 2. Cut-off scores, number of items and characteristics of the health literacy tools that were applied to estimate the prevalence of low health literacy (HL).

| Tool | Cut-off score for low HL | N. of items | Characteristics of the tool |
| --- | --- | --- | --- |
| BSAIT | ≤ 64/72 | 72 | 72 reading comprehension items |
| HALS | ≤ 275/500 | 191 | 191 health-related literacy tasks |
| HLS-EU-Q16 | ≤ 33/50 or ≤ 12/16 | 16 | 16 comprehension items |
| HLS-EU-Q47 | ≤ 33/50 | 47 | 47 comprehension items |
| HLS-EU-Q86 | ≤ 33/50 | 86 | 47 comprehension items  7 literacy and numeracy items  16 social determinant items  16 lifestyle questions and self-reported questions on health services use |
| IALS tool | < 4/4 | 4 | 4 reading comprehension questions |
| METER | ≤ 34/40 | 70 | 40 reading comprehension medical terms |
|  | ≤ 18/30 |  | 30 reading comprehension non-medical terms |
| NVS | ≤ 3/6 | 6 | 6 reading comprehension and numeracy items |
| REALM | ≤ 60/66 | 66 | 66 reading comprehension and pronunciation medical terms |
| S-FHL | answering sometimes, often, or always for one or more items | 5 | 5 items on functional skills |
| SAHLPA-23 | ≤ 19/23 | 23 | 23 reading comprehension medical terms |
| SAHLSA-50 | ≤ 37/50 | 50 | 50 reading comprehension medical terms |
| SBSQ single item | answering somewhat, a little, or not at all | 1 | answering to “how confident are you filling out medical forms by yourself?” |
| SILS | > 2 | 1 | answering to “how often do you need to have someone help when you read instructions, pamphlets, or other written material from your doctor or pharmacy?” |
| TOFHLA-short | ≤ 66/100 | 40 | 36 reading comprehension items  4 numeracy items |
| TOFHLA | ≤ 74/100 | 67 | 50 reading comprehension items  17 numeracy items |
| BSAIT: Basic Skill Assessment Initial Test. HALS: Health Activities Literacy Scale. HLS-EU-Q: European Health Literacy Survey Questionnaire. IALS: International Adult Literacy Survey. METER: Medical Term Recognition Test. NVS: Newest Vital Sign. REALM: Rapid Estimate of Adult Literacy in Medicine. S-FHL: Scale for Functional Health Literacy. SAHLPA: Short Assessment of Health Literacy in Portuguese Adults. SAHLSA: Short Assessment of Health Literacy for Spanish Adults. SBSQ: Set of Brief Screening Questions. SILS: Single Item Literacy Screener. TOFHLA: Test of Functional Health Literacy in Adults. | | | |

Supplementary Table 3. Full list of articles included in the proportion meta-analysis quantifying the prevalence of low health literacy in European Union Member States.

| **First Author,**  **year** | **Country** | **Tool** | **Target population** | **Mean or median**  **age** | **Prevalence (n/N)** | **Quality assessment**  **(Newcastle Ottawa Scale)** | | | |
| --- | --- | --- | --- | --- | --- | --- | --- | --- | --- |
|  |  |  |  |  |  | **S** | **C** | **O** | **Final score** |
| Alves, 2018 | Portugal | NVS | General population |  | 1257/1624 | 5 | 2 | 3 | 10 |
| Berens, 2016 | Germany | HLS-EU-Q47 | General population | 15-29 | 177/375 | 4 | 2 | 2 | 8 |
|  |  |  |  | 30-45 | 231/490 |  |  |  |  |
|  |  |  |  | 46-64 | 341/618 |  |  |  |  |
|  |  |  |  | 65-99 | 306/462 |  |  |  |  |
| Biasio, 2017 | Italy | METER | General population, medical students | 21.7 | 20/64 | 2 | 0 | 3 | 5 |
|  |  |  | General population,  non-medical students | 24.3 | 142/160 |  |  |  |  |
| Biasio, 2018 | Italy | METER | General population | 53.9 | 250/305 | 4 | 0 | 2 | 6 |
|  |  | SILS |  | 53.9 | 152/305 |  |  |  |  |
| Bonaccorsi, 2017 | Italy | SILS | General population | 51 | 58/174 | 4 | 2 | 3 | 9 |
|  |  | NVS |  |  | 65/174 |  |  |  |  |
| Bonaccorsi, 2019 | Italy | NVS | General population | 53.3 | 163/452 | 4 | 2 | 3 | 9 |
| Brangan, 2018 | Croatia | NVS | General population | 63.5 | 58/100 | 4 | 0 | 3 | 7 |
| Brosnan, 2012 | Ireland | REALM | Chronic disease patients | 38 | 11/40 | 3 | 0 | 2 | 5 |
| Castro-Sanchez, 2018 | Spain | NVS | General population | 32.3 | 51/119 | 5 | 2 | 3 | 10 |
|  |  | SAHLSA-50 |  |  | 17/119 |  |  |  |  |
|  |  | SILS |  |  | 56/119 |  |  |  |  |
| Cavaco, 2012 | Portugal | SAHLSA-50 | General population | 51 | 11/53 | 2 | 0 | 2 | 4 |
| Coughlan, 2012 | Ireland | REALM | General population | 61.5 | 38/199 | 4 | 0 | 3 | 7 |
| Cox, 2011 | United Kingdom | SBSQ single item | Oncology patients | 64 | 24/127 | 5 | 0 | 2 | 7 |
|  |  | REALM |  |  | 11/127 |  |  |  |  |
| Dani, 2007 | United Kingdom | REALM | General population | 67 | 30/54 | 5 | 0 | 2 | 7 |
| Diederichs, 2018 | Germany | HLS-EU-Q16 | Chronic disease patients, males | 58.6 | 566/1355 | 4 | 2 | 2 | 8 |
|  |  |  | General population, males | 58.6 | 1798/5352 |  |  |  |  |
|  |  |  | General population, females | 60.1 | 2169/6495 |  |  |  |  |
|  |  |  | Chronic disease patients, females | 60.1 | 440/942 |  |  |  |  |
| Duggan, 2014 | Ireland | REALM | General population | 31.2 | 62/404 | 5 | 2 | 3 | 10 |
| Efthymiou, 2017 | Greece | HLS-EU-Q16 | General population |  | 58/107 | 4 | 0 | 1 | 5 |
| Eronen, 2018 | Finland | HLS-EU-Q16 | General population | 75 | 106/292 | 4 | 2 | 2 | 8 |
| Espanha, 2016 | Portugal | HLS-EU-Q47 | General population |  | 1052/2104 | 5 | 0 | 1 | 6 |
| Fernandez-Silva, 2018 | Spain | HLS-EU-Q47 | Chronic disease patients | 65.4 | 84/103 | 4 | 2 | 2 | 8 |
| Fransen, 2011 | The Netherlands | REALM | Chronic disease patients, diabetes type 2 | 59.7 | 21/85 | 4 | 0 | 2 | 6 |
|  |  | REALM | Chronic disease patients, coronary artery diseases |  | 35/199 |  |  |  |  |
|  |  | NVS | Chronic disease patients, coronary artery diseases |  | 151/199 |  |  |  |  |
|  |  | NVS | Chronic disease patients, diabetes type 2 |  | 74/84 |  |  |  |  |
| Gabel, 2019 | Denmark | HLS-EU-Q16 | General population | 63.5 | 3606/7142 | 5 | 2 | 2 | 9 |
| Halleberg Nyman, 2018 | Sweden | S-FHL | General population | 47 | 277/704 | 4 | 0 | 2 | 6 |
| Heyckendorff Lilholt, 2016 | Denmark | TOFHLA | Chronic disease patients | 70.1 | 26/60 | 3 | 2 | 3 | 8 |
| Husson, 2015 | The Netherlands | SBSQ single item | Oncology patients | 70 | 224/1626 | 5 | 2 | 2 | 9 |
| Ibrahim, 2008 | United Kingdom | BSAIT | Chronic disease patients | 64 | 96/300 | 3 | 0 | 3 | 6 |
|  |  | REALM |  |  | 58/58 |  |  |  |  |
| Jordan, 2015 | Germany | HLS-EU-Q16 | General population |  | 2141/4845 | 4 | 2 | 2 | 8 |
| Juul, 2018 | Denmark | HLS-EU-Q16 | Chronic disease patients | 64 | 67/181 | 3 | 2 | 2 | 7 |
| Kobayashi, 2014 | United Kingdom | IALS | General population | 67.5 | 823/3087 | 4 | 2 | 3 | 9 |
| Kobayashi, 2016 | United Kingdom | IALS | General population | 63.3 | 1217/4345 | 4 | 2 | 3 | 9 |
| Korsbakke Emtekaer Haeseum, 2015 | Denmark | TOFHLA | Chronic disease patients | 68.7 | 19/42 | 4 | 0 | 2 | 6 |
| Koster, 2015 | The Netherlands | NVS | General population | 56.3 | 468/894 | 4 | 2 | 3 | 9 |
| Lennerling, 2018 | Sweden | NVS | Chronic disease patients | 53.5 | 23/109 | 4 | 0 | 3 | 7 |
| Mackey, 2018 | Ireland | NVS | General population, chronic pain group | 48.5 | 71/131 | 5 | 2 | 3 | 10 |
|  |  |  | General population, control group | 49.8 | 64/131 |  |  |  |  |
| McKenna, 2017 | Ireland | HLS-EU-Q47 | General population | 59 | 17/26 | 3 | 0 | 2 | 5 |
| Nafradi, 2018 | Hungary | NVS | General population | 46.5 | 122/301 | 3 | 2 | 3 | 8 |
| Ousseine, 2018 | France | SILS | Chronic disease patients | 70 | 160/269 | 4 | 2 | 2 | 8 |
| Paiva, 2014 | Portugal | METER | General population, physicians | 32.3 | 0/53 | 4 | 2 | 3 | 9 |
|  |  |  | General population, health researchers | 29.6 | 3/45 |  |  |  |  |
|  |  |  | General population,  other researchers | 43.8 | 5/50 |  |  |  |  |
|  |  |  | General population | 45.6 | 63/101 |  |  |  |  |
| Paiva, 2017 | Portugal | NVS | General population, health researchers | 28 | 5/45 | 4 | 2 | 3 | 9 |
|  |  |  | General population,  other researchers | 48.5 | 4/50 |  |  |  |  |
|  |  |  | General population | 42 | 82/101 |  |  |  |  |
|  |  |  | General population, physicians | 30 | 0/53 |  |  |  |  |
| Palumbo, 2016 | Italy | HLS-EU-Q86 | General population |  | 543/1000 | 5 | 1 | 3 | 9 |
| Pires, 2018 | Portugal | SAHLSA-23 | General population | 38.7 | 257/484 | 4 | 2 | 3 | 9 |
| Protheroe, 2015 | United Kingdom | NVS | General population | 48.7 | 505/972 | 4 | 2 | 3 | 9 |
| Puente-Maestu, 2016 | Spain | SAHLSA-50 | Chronic disease patients | 68 | 174/296 | 4 | 2 | 3 | 9 |
| Rolová, 2018 | Czech Republic | HLS-EU-Q47 | Chronic disease patients | 46.1 | 50/113 | 5 | 0 | 2 | 7 |
| Rouquette, 2018 | France | HLS-EU-Q6 | General population | 53 | 234/ 317 | 3 | 0 | 2 | 5 |
|  |  | HLS-EU-Q16 |  |  | 132/317 |  |  |  |  |
| Rowlands, 2013 | United Kingdom | REALM | Chronic disease patients | 70 | 106/687 | 5 | 2 | 3 | 10 |
| Sahm, 2012 | Ireland | REALM | General population,  from community and hospital |  | 257/1339 | 4 | 0 | 3 | 7 |
|  |  | TOFHLA-40 | General population,  from hospital |  | 59/420 |  |  |  |  |
|  |  | REALM | General population,  from hospital |  | 92/420 |  |  |  |  |
|  |  | NVS | General population,  from community and hospital |  | 766/1339 |  |  |  |  |
| Salm, 2018 | Germany | HLS-EU-Q16 | General population | 59 | 783/2000 | 5 | 2 | 2 | 9 |
| Santesmases-Masana, 2017 | Spain | HLS-EU-Q47 | Chronic disease patients | 77.9 | 253/318 | 4 | 2 | 2 | 8 |
| Schaeffer, 2017 | Germany | HLS-EU-Q47 | General population |  | 1073/2000 | 4 | 2 | 2 | 8 |
| Sorensen, 2015 | Austria | HLS-EU-Q86 | General population |  | 552/979 | 5 | 2 | 2 | 9 |
|  | Ireland |  |  |  | 384/959 |  |  |  |  |
|  | Germany |  |  |  | 484/1045 |  |  |  |  |
|  | The Netherlands |  |  |  | 285/993 |  |  |  |  |
|  | Greece |  |  |  | 447/998 |  |  |  |  |
|  | Poland |  |  |  | 411/921 |  |  |  |  |
|  | Bulgaria |  |  |  | 574/925 |  |  |  |  |
|  | Spain |  |  |  | 568/974 |  |  |  |  |
| Strijbos, 2018 | The Netherlands | NVS | Chronic disease patients | 69.9 | 154/202 | 4 | 2 | 3 | 9 |
| Sukis, 2017 | Lithuania | HLS-EU-Q47 | General population | 25 | 302/912 | 4 | 2 | 2 | 8 |
| Taylor, 2016 | United Kingdom | SILS | Chronic disease patients, kidney disease wait-listed | 50 | 270/1859 | 5 | 2 | 3 | 10 |
|  |  |  | Chronic disease patients, kidney disease incident dialysis | 58 | 481/2463 |  |  |  |  |
|  |  |  | Chronic disease patients, kidney disease incident transplant | 50 | 250/2051 |  |  |  |  |
| Tiller, 2015 | Germany | HLS-EU-Q16 | General population | 69.9 | 279/1033 | 4 | 2 | 2 | 8 |
| Van der Heide, 2013 | The Netherlands | HALS | General population | 46.7 | 2769/5136 | 4 | 2 | 2 | 8 |
| Van Schaik, 2017 | The Netherlands | NVS | Chronic disease patients | 56 | 103/199 | 4 | 0 | 3 | 7 |
|  |  | REALM |  |  | 34/198 |  |  |  |  |
| Vandenbosch, 2016 | Belgium | HLS-EU-Q16 | General population | 55.8 | 3958/9617 | 5 | 2 | 2 | 9 |
| Verkissen, 2014 | The Netherlands | SBSQ single item | Oncology patients | 62.8 | 33/265 | 4 | 2 | 2 | 8 |
| Von Wagner, 2007 | United Kingdom | TOFHLA | General population | 47.6 | 82/719 | 5 | 2 | 3 | 10 |
| Wangdahl, 2014 | Extra- EU Countries | HLS-EU-Q16 | Refugees |  | 176/285 | 3 | 2 | 2 | 7 |
|  |  | S-FHL |  |  | 316/396 |  |  |  |  |
| Wangdahl, 2018 | Extra- EU Countries | HLS-EU-Q16 | Refugees |  | 333/494 | 3 | 2 | 2 | 7 |
| Zotti, 2017 | Italy | NVS | Oncology patients | 54 | 100/245 | 4 | 0 | 3 | 7 |
|  |  | SILS |  |  | 103/245 |  |  |  |  |
| S: Selection. C: Comparability. O: Outcome.  Tool (in alphabetic order). BSAIT: Basic skill Assessment Initial Test. HALS: Health Activities Literacy Scale. HLS-EU-Q: European Health Literacy Survey Questionnaire. IALS: International Adult Literacy Survey. METER: Medical Term Recognition Test. NVS: Newest Vital Sign. REALM: Rapid Estimate of Adult Literacy in Medicine. S-FHL: Scale for Functional Health Literacy. SAHLPA: Short Assessment of Health Literacy in Portuguese Adults. SAHLSA: Short Assessment of Health Literacy for Spanish Adults. SBSQ: Set of Brief Screening Questions. SILS: Single Item Literacy Screener TOFHLA: Test of Functional Health Literacy in Adults. | | | | | | | | | |
